# Enhancing a nutrition and self-management: An intervention program via teletherapy for teenager with ADHD. A pilot case study

**DOI:** 10.1192/j.eurpsy.2021.2089

**Published:** 2021-08-13

**Authors:** T. Ahmed, E. Salem

**Affiliations:** 1 Psychology And Speech-language Pathology, COSMOMID.CA Clinic. Kuwait, Salmiya, Kuwait; 2 Psychology, Ain Shams University, Cairo, Egypt

**Keywords:** Nutrition, Self-management, Teletherapy, ADHD

## Abstract

**Introduction:**

Several ADHD teenagers had difficult behavioral problems during countries closing down due to Covid-19 pandemic. One of these negative outcomes that parents cannot control children’s behavior toward desired unhealthy food and the impulsive consequences. It was a great opportunity to convention a teletherapy program as a tool of intervention seeking for help to reduce uncontrolled self- management and nutrition, which may affect all sorts of childhood growth, development, health and behavior. Furthermore, it can affects daily life and academic success.

**Objectives:**

We tried through our study to enhance the teletherapy as a therapeutic tool, during the first and second phase of Covid-19 pandemic, trying to help parents and patient to overcome the impulsive behavior by using a specific therapy technique based on nutrition and behavioral therapy

**Methods:**

Our case study is a young girl aged 12:4 Yrs. In middle bilingual Arabic/ American School. The therapeutic program designed via teletherapy program using multi-media and thru multi phases sessions, to increase focus attention, emotional control and reduce impulsivity.

**Results:**

The outcomes of the enhancing nutrition and behavior teletherapy program, showed significant improvement for the specific goal. Sensible change in the girl’s impulsive behavior, more focusing, emotional control and more accepting about health nutrition habits.

**Conclusions:**

The important finding that intensive, focused nutation and self-management techniques provided via teletherapy as solitary program brought benefits to individual’s, family and reduced impulsivity outcomes. In addition, family education to become an expert at learning simple techniques in daily life can brining a sense of pleasure for long life wellbeing.

**Disclosure:**

No significant relationships.

